# Distribution and Structure of DM2 Repeat Tract Alleles in the German Population

**DOI:** 10.3389/fneur.2018.00463

**Published:** 2018-06-19

**Authors:** Alexis S. Mahyera, Tamara Schneider, Birgit Halliger-Keller, Katja Schrooten, Eva-Maria Hörner, Simone Rost, Wolfram Kress

**Affiliations:** Institute of Human Genetics, Julius-Maximilians-University of Würzburg, Würzburg, Germany

**Keywords:** DM2, intergenerational contraction, *de novo* expansion, premutation, penetrance, prevalence

## Abstract

Autosomal dominant inherited Myotonic dystrophy type 1 and 2 (DM1 and DM2) are the most frequent muscle dystrophies in the European population and are caused by repeat expansion mutations. For Germany cumulative empiric evidence suggests an estimated prevalence of DM2 of roughly 9 in 100,000, therefore being as prevalent as DM1. In DM2, a (CCTG)_n_ repeat tract located in the first intron of the *CNBP* gene is expanded. The CCTG repeat tract is part of a complex repeat structure comprising not only CCTG tetraplets but also repeated TG dinucleotides and TCTG tetraplet elements as well as NCTG interruptions. Here, we provide the distribution of normal sized alleles in the German population, which was found to be highly similar to the Slovak population. Sequencing of 34 unexpanded healthy range alleles in DM2 positive patients (heterozygous for a full expansion) revealed that the CCTG repeat tract is usually interrupted by at least three tetraplets which according to current opinion is supposed to render it stable against expansion. Interestingly, only the largest analyzed normal allele had 23 uninterrupted CCTGs and consequently could represent an instable early premutation allele. In our diagnostic history of DM2 cases, a total of 18 premutations were detected in 16 independent cases. Here, we describe two premutation families, one with an expansion from a premutation allele and the other with a contraction of a full expansion down to a premutation allele. Our diagnostic results support the general assumption that the premutation range of unstable CCTG stretches lies obviously between 25 and 75 CCTGs. However, the clinical significance of premutation alleles is still unclear. In the light of the two described families we suggest incomplete penetrance. Thus, as it was proposed for other repeat expansion diseases (e.g., Huntington's disease), a fluid transition of penetrance is more likely rather than a clear cut CCTG number threshold.

## Introduction

Myotonic dystrophy type 2 (DM2; PROMM; Ricker syndrome, MIM 602668) is an autosomal dominant multisystemic neuromuscular disorder that occurs in adults (1, 2). Although DM2 and DM1 share the same core features (progressive muscle weakness and atrophy, myotonia, cataracts) as well as additional symptoms like muscle pain, cardiac arrhythmias, endocrinologic disturbances and hypersomnia, apparent differences exist. The disease course is usually more variable and milder in DM2 with paucisymptomatic cases (3). Brain involvement is rare, there is no congenital form as well as evident anticipation, but myalgic pain is a frequent symptom and muscle weakness is mainly proximal. The frequency of DM2 is highest in Middle Europe.

DM2 is caused by the expansion of an unstable (CCTG)_n_ repeat located in the first intron of the *CNBP* (cellular retroviral nucleic acid-binding protein) gene in the 3q21.3 chromosomal region (4). Like in DM1 the underlying pathophysiology is a toxic RNA gain-of-function mechanism. The pre-mRNA contains the repeat expansion and remains unprocessed (5). It disrupts cellular pathways like RNA splicing, localization and translation through sequestering of important RNA binding proteins. In muscle fibers insoluable ribonuclear foci are formed. As a result, developmentally inappropriate protein isoforms are expressed in adult tissue.

The complex *CNBP* repeat tract is generally described as (TG)_n_(TCTG)_n_(CCTG)_n_. Normal alleles as a rule have <25 copies of the decisive CCTG repeat, whereas expanded alleles have as many as 75–11,000 copies (4). However, the clinical significance of the presumed premutation repeat range between 25 and 75 CCTGs is still unclear and subject of discussion.

In normal sized alleles the CCTG repeat tract is usually found to be interrupted by one or more tetraplets [(NCTG)_n_], resulting in more or less stability (4, 6, 7). Consequently, the combined repeat tract in those healthy range alleles can be described by five distinct repetitive motifs: (TG)_v_(TCTG)_w_(CCTG)_x_(NCTG)_y_(CCTG)_z_. In contrast, unstable expanded alleles obviously have a pure CCTG stretch without the NCTG interruption making them more susceptible to strand slippage and unequal crossing over processes in cell division (6) and favoring unusual secondary structures (8).

The composition of the complex repeat motif and allele frequency of normal and expanded alleles has already been studied in the Slovak and American population (6, 7). So far, no equivalent analysis of the *CNBP* repeat motif has been conducted for the German population. In the present report, an estimate of the DM2 prevalence in Germany is given and a statistical analysis of the normal allele length frequency and distribution was performed. Alleles of the most frequent repeat lengths (*n* = 31) as well as of the longest normal alleles (*n* = 3) were sequenced to determine the detailed motif composition and its variance within the German population. Furthermore, we present two small families, one with a transgenerational contraction into the premutational range and another with a transgenerational expansion of a premutation allele to a full expansion.

## Materials and methods

### Fragment analysis data

**Figure 1:**
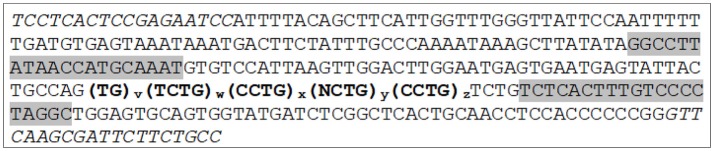
The combined *CNBP* repeat tract and its surrounding specific sequence with indicated primer binding sites for PCR amplification. Primers for fragment analysis are highlighted in gray and indicate the fragment analysis amplicon. Primer binding sites for sequence analysis from Radvanszky et al. (7) are indicated in italics. The combined repeat tract is denoted in bold. For statistical analysis, the specific combined repeat tract lengths were calculated by subtracting 92 bp of specific sequence surrounding the *CNBP* repeat (68 bp upstream and 24 bp downstream the bold repeat tract) from the measured amplicon length by fragment analysis.

Fragment analysis by capillary electrophoresis was used to identify the length of unexpanded normal alleles. Data on the repeat length from 739 DM2 negative German probands were collected as a result of routine diagnostic fragment analysis, and statistically analyzed for allele frequencies. Patients' data were anonymized. Genomic DNA was isolated from leukocytes and extracted using standard laboratory procedures. Fragment analysis was performed as previously described (4) with minor modifications. Briefly, the method comprises PCR amplification of the *CNBP* repeat sequence, using a fluorescently marked 6-F-primer (DM2-Cl3N58-F) 5′-GGC CTT ATA ACC ATG CAA ATG-3′, binding 47 bp upstream of the TG repeat sequence. The reverse primer (DM2-Cl3N58-R) 5′ GCC TAG GGG ACA AAG TGA GA 3′ binds directly after the last single TCTG tetraplet following the repetitive sequence (Figure 1). Up to 40 CCTG repeats can be amplified with this method. The PCR cycle program consists of the following five steps: Step 1: 95°C (5 min), step 2: 95°C (30 s), step 3: annealing at 56°C (30 s), step 4: elongation at 72°C (30 s), and step 5: final elongation at 72°C (3 min). Steps 2–4 were repeated in 30 rounds. After DNA amplification, 15 μl Hi-Di™ formamide (Thermo Fischer) and 0.5 μl of the MapMarker® 1000 X-Rhodamine (BioVentures) was added to 1 μl of the PCR product and followed by capillary electrophoresis on an Applied Biosystems 3130/3130xl Genetic Analyzer. Genemapper 4.0 was used to analyze the data. For statistical analysis, the combined repeat tract lengths of each allele observed in fragment analysis were calculated by subtracting 92 bp of specific sequence surrounding the *CNBP* repeat from the measured amplicon length. For illustration the *CNBP* repeat sequence including the combined repeat tract length and primer binding sites are depicted in Figure 1. The statistical analysis of the frequencies and distribution of the different allele lengths in the German population was done with MS Excel (Office Suite 2016). Please note, that the normal alleles of DM2 positive were not included in the statistical analysis of healthy range repeat sizes. Confirmation of DM2 positive patients was done by RP-PCR (repeat primed PCR) and Southern blotting in routine diagnostics [protocol in (9, 10)].

### Sanger sequencing

Sanger sequencing was conducted in order to characterize *CNBP* repeat motif compositions of the three most frequent allele lengths as well as the longest normal alleles in the German population. Since the length and composition of the repeat motif varies on each allele, Sanger sequencing of blood DNA is challenging in most cases due to overlapping and shifted sequences from two different alleles which impedes a proper sequence read out. Therefore, instead of sequencing probands with homozygous or heterozygous allele lengths in the normal range, 34 DM2-positive patients with full expansion on one allele were sequenced. By reducing PCR elongation time to a minimum of only 2 s, amplification was strongly biased toward the short normal repeat allele rather than the expanded allele. The application of a discriminating PCR protocol allowed the detailed determination of a single allele's sequence avoiding co-amplification of the expanded allele and thereby any overlapping background from a second allele. The PCR primers for sequencing of healthy range length alleles have previously been described by Radvanszky et al. (7). Their binding sites within the *CNBP* sequence are depicted in Figure 1 in italics. In detail, the applied touchdown PCR protocol comprised the following annealing temperature steps: 62°C (30 s, 2 cycles), 59°C (30 s, 2 cycles), and 56°C (30 s, 30 cycles), each followed by an elongation step for only 2 s at 72°C, and a final elongation step of 5 min. PCR products were purified using ExoSAP-IT (Affymetrix, Santa Clara, CA) treatment.

In order to get an impression of the heterozygosity of the locus, 11 DM2 negative patients that appeared to be homozygous due to a single peak in fragment analysis were also sequenced with the same conditions described above.

All DNA samples were sequenced applying the BigDye Terminator v1.1 Cycle Sequencing Kit and 0.5 μg single-stranded DNA binding protein (Promega) on an Applied Biosystems 3130 Genetic Analyzer. Sequencing conditions were the following: Step 1: 96°C (2 min), step 2: 96°C (20 s), step 3: 56°C (20 s), step 4: 60°C (3 min), and step 5: 60°C (5 min). Steps 2–4 were repeated in 26 rounds. Data were analyzed with the software Chromas lite (Technelysium). All sequenced patients had given informed consent for research purposes in the DM2 field, as required by the declaration of Helsinki 2013.

## Results

### Prevalence of DM2

There are no well-documented prevalence data for DM2 except of the reports from Finland (11) and Rome Province in Italy (12), respectively. We are running one of the main diagnostic labs for neuromuscular diseases performing an estimated proportion of 75% of all tests for DM1/DM2 in Germany. From our own experience in the diagnostic lab, it is obvious that DM2 and DM1 contribute equally to the myotonic dystrophy phenotype as we detected about the same number of DM2 and DM1 patients by molecular genetic testing over many years (Table 1). Therefore, cumulative empiric evidence suggests that DM2 exhibits quite the same prevalence like DM1 in the German population. The submitters routinely send us both DM2 and DM1 samples, as is evident from the years shown in Table 1. In contrast to DM1, an indication for the test was generally given more liberally for DM2. Taking the different prevalences of DM1 from pre-molecular times (13) and making the assumption of a big phenotypical overlap due to a lack of diagnostic discriminability between DM1 and DM2 at that time, each of the two diseases should have about half of the previously stated prevalence. This would lead to an arithmetic prevalence of about 9 per 100,000 person-years or roughly 7,200 patients in Germany.

**Table 1:** Numbers of confirmed DM1 and DM2 positive patients in the period between 2004 and 2009 in the Würzburg lab.

	**DM1**			**DM2**		
**Year**	**Total**	**Positive**	**%**	**Total**	**Positive**	**%**
2004	170	110	64.7	270	115	42.6
2005	265	139	52.5	312	141	45.2
2006	233	94	40.3	314	103	32.8
2007	267	137	51.3	328	103	31.4
2008	221	102	46.2	396	106	26.8
2009	322	102	32.2	447	87	19.5

*The total number represents the number of patients that was send to the lab with the clinical diagnosis of DM1 and/or DM2, respectively. Assignment to the suspected diagnoses was done by the treating physician. The number and percentage of positive patients represents the proportion of total patients in which the diagnosis DM1 or DM2, respectively, could be confirmed in our laboratory*.

### Statistical analysis of allele length frequencies

**Figure 2:**
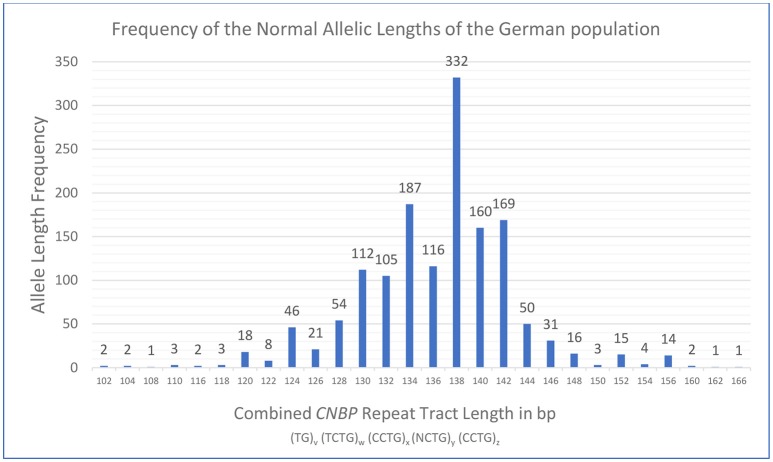
Distribution and frequency of healthy range alleles of the *CNBP* repeat in the German population obtained from analysis of 1478 DM2 negative probands. Analysis revealed a range of the combined *CNBP* repeat tract lengths from 102 to 166 bp at the very extreme ends. Please note, that the longest healthy range allele observed in this study (168 bp) was found in combination with a pathological repeat expansion on the second allele in a DM2 positive patient and is therefore not part of the frequency plot of DM2 negative probands.

For this study, fragment analysis data were collected from 739 probands of presumed German origin in order to define the polymorphic spectrum of DM2 repeat tract lengths in the German population. Figure 2 shows the frequency of alleles with different fragment lengths of DM2 negative probands. The combined repeat tract length was plotted against the allele frequency. In this set of samples, a unimodal distribution of 28 distinct allelic lengths is shown, ranging from 102 to 166 bp. The most frequent alleles in the German population consisted of 134, 138, 140, and 142 bp. Notably, while screening the normal alleles of DM2 positive patients in order to sequence the largest unexpanded alleles, we detected the longest allele at 168 bp, which is only 2 bp more than the longest allele (166 bp) found in DM2 negative patients (Table 2).

**Table 2:** Composition of the combined repeat tract [(TG)_v_ (TCTG)_w_ (CCTG)_x_ (NCTG)_y_ (CCTG)_z_] of non-expanded, normal alleles in 34 DM2 positive patients determined by sequencing analysis.

**Combined repeat size (bp)**	**(TG)_v_**	**(TCTG)_w_**	**(CCTG)_x_**	**(NCTG)_y_**	**(CCTG)_z_**
	**v**	**w**	**x**	**y**	**z**
134	21	8	5	3	7
	21	8	5	3	7
	21	8	5	3	7
	21	8	5	3	7
	19	9	5	3	7
	19	9	5	3	7
	19	9	5	3	7
	19	9	5	3	7
	17	10	5	3	7
	17	10	5	3	7
	21	7	6	3	7
	19	8	6	3	7
138	23	8	5	3	7
	23	8	5	3	7
	21	9	5	3	7
	21	9	5	3	7
	21	9	5	3	7
	21	9	5	3	7
	21	9	5	3	7
	21	9	5	3	7
	21	9	5	3	7
	21	9	5	3	7
	19	10	5	3	7
	19	10	5	3	7
	19	10	5	3	7
	23	7	6	3	7
	21	8	6	3	7
142	23	9	5	3	7
	21	10	5	3	7
	21	10	5	3	7
	25	7	6	3	7
152	22	8	8	5	6
156	22	9	8	5	6
168	18	10	23	–	–

*Indices v, w, x, y, and z denote the number of the specific repeat elements. While y and z were constant (y = 3, z = 7) throughout all 31 patients with repeat sizes of 134, 138, or 142 bp, x was found to be binary with x = 5 being about five times more frequent than x = 6. In contrast, v and w were highly variable with v ranging from 17 to 25 and w ranging from 7 to 10 in all 34 patients. The interruption of the two CCTG tracts by (NCTG)_y_ was found to comprise three tetraplets in the high abundance alleles and five in two of the three large normal alleles (152 and 156 bp). The largest allele of 168 bp showed no interruption (y = 0) but 23 contiguous CCTGs*.

### Expansion and contraction of probably pathologic alleles in two small families

**Figure 3:**
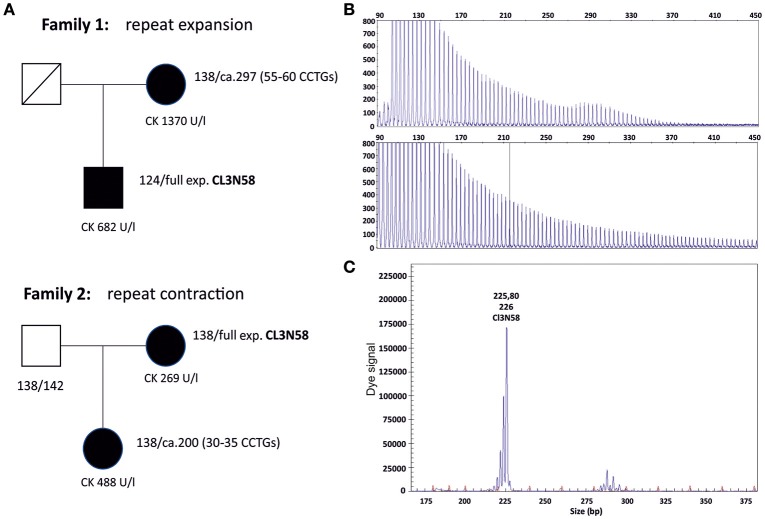
**(A)** Pedigrees of two families with expansion and contraction events in meiosis. Family 1 shows a CCTG expansion from a premutation allele to a full expansion, family 2 a CCTG contraction from a full expansion to a premutation allele. Lengths of the combined repeat tract of both alleles are given in base pairs next to the patients. Using the repeat marker CL3N59 which is in linkage disequilibrium with the *CNBP* repeat marker (CL3N58), the contraction and expansion events could be linked to the respective expanded allele (data not shown). **(B)** Electropherograms of the repeat primed PCR (RP-PCR) of the affected members of family 1. The RP-PCR patterns are compatible with a pure CCTG stretch without interruptions by non CCTG tetraplets. **(C)** Fragment analysis of the contracted allele in family 2 (daughter). The premutation allele is represented by a wave of peaks. The fragment length (PCR product length) is ~290 bp, corresponding to a combined repeat tract of about 200 bp. All expansions were controlled by Southern blotting (data not shown).

During our diagnostic procedures we observed the expansion of a (CCTG)_~55_ allele to a full expansion (family 1) and the contraction of a full expansion to a (CCTG)_~30_ allele (family 2, Figure 3). Both small expansion alleles are in the range of putative premutations (25–75 CCTGs) (4). The profiles of the repeat primed PCR (RP-PCR) analysis provide an approximate number of uninterrupted CCTGs in family 1. Phenotypic description of the two patients harboring the small expansion alleles were compatible with DM2: in family 1, symptoms of the mother with the small expansion fit the diagnosis DM2 including proximal weakness, myotonia and typical discharges in EMG. In family 2, the patient (daughter) with the contraction to about 30 CCTGs had a “mild myopathy” at the time of the test. Sixteen further premutation alleles ranging from about 26 to 55 CCTGs with an uninterrupted pattern in RP-PCR were observed in the last few years. The phenotype of the probands is in most cases not characteristic enough to make a clear diagnostic decision in favor of DM2.

### Sequencing analysis

The composition of the complex repeat motive of normal length alleles in the German population was analyzed by Sanger sequencing of DNAs from 34 DM2 patients with repeat expansion determined by routine diagnostic fragment analysis. Thirty one of the 34 DM2 positive patients were specifically selected because they possessed one of the three most frequent repeat lengths (136, 138, or 142 bp). The remaining three DNAs were derived from those three DM2 patients with a full expansion that revealed the three longest healthy range alleles found in our patient cohort (152, 156, and 168 bp). Table 2 summarizes the compositions of specific alleles in DM2 positive patients. Moreover, eleven DM2 negative probands that appeared to be homozygous due to a single allele in fragment analysis were selected for sequencing to get an estimation of the real homozygosity frequency (data not shown).

In case of the 31 patients with the high abundance alleles (136, 138, or 142 bp), in all of the analyzed healthy range alleles of the *CNBP* repeat the CCTG tract was interrupted by three tetraplets (NCTG)_3_: GCTG CCTG TCTG. The last CCTG repeat unit always comprised 7 CCTGs whereas the first CCTG unit before the interrupting (NCTG)_3_ was found to vary between 5 and 6 CCTG tetraplets. Of the 31 sequenced alleles 26 revealed (CCTG)_5_(NCTG)_3_(CCTG)_7_ (84%) while only 5 alleles (16%) had 6 leading CCTG tetraplets [(CCTG)_6_(NCTG)_3_(CCTG)_7_]. In contrast to the highly stable CCTG tract, the (TG)_v_ and (TCTG)_w_ motifs at the beginning of the *CNBP* repeat stretch vary widely within the range of v = 17–25 (TG)_v_ and w = 7–10 (TCTG)_w_. The most frequent normal allele was identified to contain 9 TCTG tetraplets [(TG)_v_(TCTG)_9_(CCTG)_5_(NCTG)_3_(CCTG)_7_], with a frequency of 42% of the 31 analyzed alleles and a range of (TG)_v_ of v = 19–23.

Composition of the two longest normal alleles (152 and 156 bp) was similar to each other: (TG)_22_ (TCTG)_w_(CCTG)_8_(NCTG)_5_(CCTG)_6._ Specifically, in both alleles the interruption comprised five tetraplets: GCTG CCTG TCTG CCTG TCTG. The allele of 168 bp was found to be an exception with 23 uninterrupted CCTG tetraplets (Table 2).

Interestingly, only 2 of the 11 DM2-negative probands that appeared to be homozygous in the fragment analysis were indeed homozygous while the other 9 probands had repeat tracts with an identical length but a different motif composition where particularly TG and TCTG repeats varied widely. One of these true homozygous patients was homozygous for the allele (TG)_21_(TCTG)_9_(CCTG)_5_(NCTG)_3_(CCTG)_7_ (138bp) while the other for (TG)_21_ (TCTG)_10_(CCTG)_5_(NCTG)_3_(CCTG)_7_ (142bp)_._ Another patient with homozygous allele lengths but heterozygous motif composition revealed a rare allele (TG)_21_ (TCTG)_7_(CCTG)_6_(NCTG)_3_(CCTG)_9_ where the second CCTG motif had 9 instead of the constant number of 7 CCTGs (data not shown).

## Discussion

### Prevalence data

The estimation of DM2 prevalence in Germany of about 9 in 100,000 people is equivalent to findings in the Italian population (12). For the German population it is quite clear from routine DM diagnostics that DM1 and DM2 have the same prevalence, but this varies in other countries. Moreover, there is increasing evidence from other reports that the DM2 prevalence in Europe is at least as high as that of DM1 or might be even higher (7, 11, 14). The reason of this relatively high prevalence in Northern Europe may be due to a founder effect (15) which was recently proposed as originating in the region of Slesia (3).

### Distribution and frequencies of healthy range *CNBP* repeat alleles

The distribution and the size range of short healthy range alleles of the *CNBP* repeat tract seem to be essentially similar among European populations. Comparing the German population's combined tract lengths [(TG)_v_(TCTG)_w_(CCTG)_x_(NCTG)_y_(CCTG)_z_] with those of the Slovak population underscores this hypothesis. The distribution of most healthy range alleles was found to be almost identical to the statistical results of the Slovak population (7): the range of normal alleles within 95 Slovak probands or 190 alleles was 118–156 bp with only one outlier of 184 bp with questionable clinical significance. In the bigger German cohort of 739 probands (or 1,478 alleles) most alleles were found to be situated in the same range between 120 and 156 bp. Due to the higher number of cases extreme ends of the distribution even extended from 102 to 166 bp including a few rare alleles.

The three most frequent allele lengths among the German population consisted of 138, 134, and 142 bp (most frequent to third-most frequent). Radvanszky et al. identified 138 bp as the most common tract length, which was found to be the most frequent in the German population, too. The second-most frequent allele length of 134 bp among the German population was in third place in the Slovak population, whereas the third-most frequent tract length of 142 bp in the German population was at second place among the Slovak allele lengths (7). Notably, abundance of the second and third most frequent alleles was very similar to each other in both our and the Slovak population.

### Structural composition of the three most frequent alleles in the German population

The structure of the combined repeat tract is known to be highly polymorphic which is mainly attributed to the most polymorphic part, the (TG)_v_(TCTG)_w_ stretch. Accordingly, sequencing analysis of probands with only one apparently homozygous allele in the fragment analysis showed that fragment length alone does not predict homozygosity *per se* due to structural differences of the complex repeat. Indeed, a monomorphic structure was rarely observed (<20%). This is in accordance with other studies where heterozygosity was found to be roughly 90% in different European populations (4, 6, 7). Sequencing of the three most frequent alleles revealed only slight variation of the first CCTG stretch with x equaling either 5 or 6. The (NCTG)_y_(CCTG)_z_ part in contrast was fully stable in all 31 probands. The most common (CCTG)_5_(NCTG)_3_(CCTG)_7_ allele accounted for 26 of the 31 alleles (84%) while only 5 (16%) belonged to the (CCTG)_6_(NCTG)_3_(CCTG)_7_ allele. However, other rare alleles are present in the German population as observed in a “homozygous” but actually heterozygous DM2 negative proband, where a (CCTG)_6_(NCTG)_3_(CCTG)_9_ allele with *z* = 9 instead of *z* = 7 was present. Our results correlate well with the observations in the Slovak population where 75.3% of probands contained the (CCTG)_5_(NCTG)_3_(CCTG)_7_ and 17.4% the (CCTG)_6_(NCTG)_3_(CCTG)_7_ allele (7) while only few other alleles could be detected. The interrupting motif (NCTG)_y_ was always found to consist of the three consecutive tetraplets (CGTG)(CCTG)(TCTG) for the three most frequent alleles which has also been previously reported by Radvanszky et al. (7) and is considered to render the allele stable against expansion (2, 7).

Notably, the structural composition of 2 of the 3 longest analyzed normal alleles (152 and 156 bp) varied only in the amount of TCTG tetraplets. The CCTG interruption (NCTG)_5_ consisted of an identical GCTG CCTG TCTG CCTG TCTG sequence in both alleles.

### Conclusions from the German cohort analysis on premutation range, repeat instability, and penetrance

It is suspected that frequent repeat tract lengths with interruptions are inherited stably in germline and somatically (2, 7). Accordingly, the CCTG tract of all 34 sequenced German alleles had an interruption of three (CGTG)_1_(CCTG)_1_(TCTG)_1_ or five (GCTG)_1_(CCTG)_1_(TCTG)_1_(CCTG)_1_(TCTG)_1_ tetraplets, respectively. Thus, the high polymorphic structure of the *CNBP* repeat is also recognizable from its interrupting tetraplets. However, uninterrupted alleles have previously been identified as being distributed over the whole size spectrum of 190 normal sized repeats (118–184 bp) and constituted 2.6% in the randomly selected Slovak individuals (7). These findings raise the question about where instability of uninterrupted alleles starts and whether a distinct instability threshold at a certain number of CCTGs can reliably be defined. Such a threshold would be expected at the upper end of the healthy repeat size range or beyond. The largest interrupted and therefore considerably stable repeat tract in Radvanszky's study encompassed 156 bp which is in accordance with our findings of a 152 and a 156 bp interrupted allele. Only few single alleles above that size could be identified in our analysis which supports a possible upper end of normal repeat tract size at around 156 bp.

The largest combined repeat tract length of presumed healthy range alleles in the German population was a 168 bp allele and was identified in a DM2 positive patient as second allele beside the expanded allele. Sequencing revealed that this rare allele has an uninterrupted stretch of 23 CCTGs but most probably does not contribute to the DM2 phenotype in the affected patient. Moreover, this CCTG stretch length is still below an estimated general instability threshold of 100–200 bp or 25–55 repeats, respectively (16). For comparison, the largest identified allele in the randomly selected Slovak individuals was a single 184 bp allele that revealed even 30 uninterrupted CCTGs according to RP-PCR. However, this allele and the 23 CCTGs allele in our study might both already represent early instable or premutation alleles. Radvanszky et al. concluded from their findings in the Slovak population that the instability threshold most likely begins moderately at 30 uninterrupted CCTGs and further increases with the length of the CCTG stretch (7). In contrast, as observed in the members of family 2, contraction of an expanded allele to ~30–35 uninterrupted CCTGs created a fully penetrant pathological allele of a size very close to healthy range alleles. The observation of an expansion and contraction from and to a fully penetrant “premutation” allele in the two families (Figure 3) shifts the potential pathological repeat range from 75 CCTGs to lower repeat values. It is worth to note that intergenerational contractions or *de novo* expansions of the *CNBP* repeat tract haven't been reported so far. The observation of transgenerational contraction events argues in favor of an unequal crossing over rather than strand slippage as a potential pathomechansim. It is not definitely clear whether these shorter uninterrupted CCTG stretches between 30 and 55 are fully penetrant, but at least in family 2 it is the case. We may face a bit of the situation in Huntington's disease: alleles with a lower number than 39 CAG repeats could be pathologic but are phenotypically not fully penetrant. Accordingly, we suggest that there may be an incomplete penetrance for premutation alleles in the range of ~25–75 uninterrupted CCTGs.

## Conclusion

Our observations in the German population lead to the assumption that alleles with a combined *CNBP* repeat tract length of around 156 bp can be considered to define the upper end of stable alleles as it represents the biggest interrupted normal allele from more than 700 probands and more than DM2 patients harboring an expansion. Premutation alleles without interruption could have originated from either an expansion event as well as from repeat contractions and can show full penetrance like in our two families 1 and 2. We therefore suspect that instability starts in the range of 25–30 uninterrupted CCTGs. According to findings for other repeat expansion diseases, like Huntington's disease, we suggest that the premutation range (25–75 CCTGs) shows incomplete penetrance which might depend on different parameters not known to date but could encompass specific changes in the repeat structure to patients' physical conditions or even environmental impacts. Collecting data from more patients with “small” DM2 expansions and known repeat tract structure as well as a follow up over time is necessary to estimate penetrance and should be a goal of future research to provide better genetic counseling for DM2 patients.

## Ethics statement

All subjects gave written informed consent in accordance with the Declaration of Helsinki.

## Author contributions

AM, TS, and WK data collection, data analysis, and data interpretation. AM, TS, WK, and SR drafting and critically revising the manuscript. BH-K, E-MH, and KS genetic testing.

### Conflict of interest statement

The authors declare that the research was conducted in the absence of any commercial or financial relationships that could be construed as a potential conflict of interest. The reviewer PM and handling Editor declared their shared affiliation.
